# Hrip1 mediates rice cell wall fortification and phytoalexins elicitation to confer immunity against *Magnaporthe oryzae*

**DOI:** 10.3389/fpls.2022.980821

**Published:** 2022-09-23

**Authors:** Vincent Ninkuu, Jianpei Yan, Lin Zhang, Zhenchao Fu, Tengfeng Yang, Shupeng Li, Beibei Li, Jiaqi Duan, Jie Ren, Guangyue Li, Xiufen Yang, Hongmei Zeng

**Affiliations:** State Key Laboratory for Biology of Plant Diseases and Insect Pests, Institute of Plant Protection, (CAAS), Beijing, China

**Keywords:** Hrip1, lignin, diterpenoids, benzoxazinoids, *Magnaporthe oryzae*, *Oryzae sativa*

## Abstract

*Magnaporthe oryzae* is a potent fungus that adversely affects rice yield. Combinatorial techniques of prevention, toxic chemicals, and fungicide are used to remedy rice blast infection. We reported the role of Hrip1 in cell death elicitation and expression of systematic acquired resistance that could potentially stifle *M. oryzae* infection. In this study, transcriptome and metabolomic techniques were used to investigate the mechanism by which Hrip1 reprogramed the transcriptome of rice seedlings to confer immunity against *M. oryzae*. Our results showed that Hrip1 induces cell wall thickening and phytoalexin elicitation to confer immunity against *M. oryzae* infection. Hrip1 activates key lignin biosynthetic genes and myeloblastosis transcription factors that act as molecular switches for lignin production. Lignin content was increased by 68.46% and more after 48 h onwards in Hrip1-treated seedlings compared to the control treatment. Further analysis of cell wall morphology using the transmission electron microscopy technique revealed over 100% cell wall robustness. Hrip1 also induced the expression of 24 diterpene synthases. These include class I and II terpene synthases, cytochrome P450 subfamilies (OsCYP76M and OsCYP71Z), and momilactones synthases. The relationship between the expression of these genes and metabolic elicitation was analyzed using ultra-performance liquid chromatography–tandem mass spectrometry. Enhanced amounts of momilactones A and B, oryzalactone, and phytocassane A and G were detected in the Hrip1-treated leaves. We also identified seven benzoxazinoid genes (BX1-BX7) that could improve rice immunity. Our findings show that Hrip1 confers dual immunity by leveraging lignin and phytoalexins for physical and chemical resistance. This study provides novel insights into the mechanisms underlying Hrip1-treated plant immunity.

## Introduction

*Magnaporthe oryzae*, the causative agent of rice blast disease, is a devastating fungus responsible for the loss of yield equivalent to the quantity of rice consumed by 60 million people annually (Bagnaresi et al., 2012; Martin-Urdiroz et al., 2016). The use of synthetic chemicals to control this fungus is harmful to the environment and, therefore, unsustainable (Wang et al., 2018). This phenomenon has attracted considerable attention in biopesticide research as an alternative with little or no adverse environmental hazards.

Plants recognize microbial molecules using cell-surface receptors to activate unique signaling cascades to aid their defense mechanisms. Two lines of immunity occur in plants. The detection of microbial-associated molecular patterns (MAMPs or PAMPs) through pattern recognition receptors evokes the first line of defense. PAMP-triggered immunity (PTI) is mediated by cell-surface receptor-like kinases (RLKs) that perceive PAMPs or MAMPs (Cheng et al., 2013). However, successful pathogens compromise PTI by secreting virulence effector proteins targeted at crucial host immune response mechanisms. Plants evoke the second line of defense against virulent effectors using intercellular nucleotide-binding domain leucine-rich repeat (NLR) proteins. NLR proteins initiate the second line of immunity (effector-triggered immunity) by recognizing effectors (Cheng et al., 2013).

After PRR perceives PAMPs, signaling activities commence, resulting in the activation of many defense arsenals such as phytoalexin production, callose deposition, and reactive oxygen species induction (Larroque et al., 2013). Accumulation of phytoalexins improves plant defense. Szatmári et al. (2014) demonstrated that phenylpropanoids enhance PTI induction against bacterial infections in tobacco plants. An aphid-derived elicitor was also reported to induce phytoalexin-deficient-3 gene for camalexin accumulation against green peach aphids (Prince et al., 2014). Although the function of phytoalexins in PTI is seldom disputed, omics techniques continue to expand the frontiers of characterized and novel defense compounds in plants. For example, Kariya et al. (2020) recently isolated *M. oryzae*–suppressive phytocassanes G and oryzalactone from rice.

Rice plants produce numerous metabolites, including phenolics, terpenes, and flavonoids. Diterpenoids are a C-20 superfamily of terpenes with antimicrobial and antifungal characteristics. The isolation of momilactone, oryzalexin, oryzalexin S, and phytocassane diterpenoids has been linked to rice blast infections (Lu et al., 2018). In addition, phenolic compounds such as lignin, cellulose, and pectin contribute to cell wall integrity (CWI) for PTI induction (Engelsdorf et al., 2018, 2019). Lignin also perverts pathogen-degrading enzymes and restricts the mobility of pathogens from infecting new cells (Shinde et al., 2017). Moreover, lignin and callose deposition block fungal haustoria (Cui et al., 2018). Benzoxazinoids (BXs) are robust phytochemicals that aid plant defense (Hu et al., 2018). Although there are no reports on BXs biosynthesis in rice, their protective and allelopathic activities in other Poaceae family members such as maize, wheat, and rye have been reported. BXs, such as 2,4-dihydroxy-1,4-benzoxazine-3-one (DIBOA_glycoside), dihydroxy-7-methoxy-1,4-benzoxazine-3-one (DIMBOA_glycoside), and 2,4,7-trihydroxy-2H-1,4-benzoxazine-3-(4H)-one (TRIBOA_glycoside) induce immunity against pests and diseases (Dick et al., 2012; Sue et al., 2021).

Elicitors are low-molecular-weight proteins, glycoproteins, lipids, and oligosaccharides derived from viruses, bacteria, and fungi (Patel et al., 2020). Fungal-derived elicitors induce defense metabolite accumulation to alter the physiological conditions of plants (Patel et al., 2020). For example, exogenous treatment of rice seedlings with MoHrip1 enhances salicylic acid elicitation against *M. oryzae* (Lv et al., 2016).

Our laboratory isolated a hypersensitive response-inducing protein (Hrip1) from *Alternaria tenuissima* that induces calcium influx, medium alkalinization, and salicylic acid-induced protein kinase. Hrip1 (GenBank accession numberHQ713431) also activates many defense genes and systemic acquired resistance after several days of treatment in tobacco leaves (Kulye et al., 2012). Hrip1 transgenic lines in *Arabidopsis thaliana* promote plant growth under stressful salt and drought conditions and confer resistance to *Botrytis cinerea* (Peng et al., 2015). The current study shows that Hrip1 also mediates immunity against rice blast fungi through cell wall enhancement and phytoalexin accumulation.

## Experimental procedure

### Plant growth and elicitor preparation

Rice cv. Nipponbare (*Oryza sativa* spp. japonica) seeds were surface-sterilized in a 5% NaClO solution for 10 min and rinsed seven times with double-distilled water (ddH_2_O). The seeds were spread on soaked Whatman filter paper, placed in a 30 mm Petri dish, and incubated in a growth chamber to sprout. The growth chamber was set at 30°C under light and 25°C in the dark under a 12/12 h photoperiod. Seedlings were transplanted into nutrient-rich soil after 5 days and grown in a greenhouse under the same conditions. All experimental plants were treated as below at the third-leaf stage.

The Hrip1 gene was amplified and inserted into the pPICZαA vector. The recombinant plasmid (pPICZαA-Hrip1) was transformed into *Escherichia coli* and electroporated into the competent yeast cells. A volume of 5 μl of pPICZαA-Hrip1 was spread on a YPD plate containing Zeocin for selecting positive clones. A single yeast cell was used to inoculate 50 ml YPD and shaken in an incubator at 200 rpm and 30°C for 48 h to attain an OD600 = 2–6 in 48 h. Yeast genomic DNA was isolated from 2 ml of the yeast using the yeast genomic DNA extraction kit from Solarbio Biotechnology Company (Beijing). The Hrip1 DNA was then amplified using PCR primer pairs (Forward primer: GCTCCTACTACTATGAACGGCC Reverse primer: GCACTGAGGCAAGTTACAGAC). A volume of 50 μl PCR mix consisting of H_2_O (35 μl), dNTPs (5 μl), MgSO_4_ (5 μl), 1.5 μl each of forward and reverse primers, 1 μl each of KOD-Plus and Hrip1 DNA was prepared. A thermocycler condition for 35 cycles was set at 94°C predenaturation for 2 min, and denaturation for 15 s. Also 68°C was set for 30 s annealing and 1 min extension. The Hrip1 band was purified using the EasyPure quick gel extraction kit purchased from Transgen Biotechnology Company (Beijing). An aliquot of 20 μl was taken into a clean microcentrifuge tube for commercial sequencing at Sangon Biotechnology Company (Beijing). The Hrip1 sequence alignment was performed using the DNAMAN software.

After the correct sequences were determined, 1,000 ml of yeast nitrogen base (YNB) medium was inoculated with 10 μl of the recombinant pPICZαA-Hrip1 and shaken as described for 4 days. After centrifugation at 3,000 × *g*, the cells were resuspended in 100 ml YNB medium (without glycerol), incubated under the same conditions, and induced every 24 h with 500 μl of 100% methanol. The supernatant was collected after centrifugation at 10,000 × *g* at room temperature for 15 min and filtered through a 45 μm Millex-HV syringe filter. Hrip1 secreted protein was then subjected to affinity chromatography on a HiTrap column (GE Healthcare, Waukesha, WI, United States) and ion-exchange chromatography on a Cytiva HiPrep 26/10 Desalting column. The molecular weight of the protein was confirmed by loading 15 μl into 12% sodium dodecyl sulfate–polyacrylamide gel electrophoresis (SDS-PAGE). The protein concentration was analyzed using the Solar Bio BCA protein assay kit (Beijing, China). A volume of 30 μm Hrip1 was reconstituted from the stock protein to treat rice seedlings by spraying at the third-leaf stage. Control plants were sprayed with 30 μM Tris–HCl buffer, pH8.0. Triplicate samples of leaves were obtained from both Hrip1-treated (HT), and control buffer-treated (CB) plants after 6, 12, 24, and 48 h for RNA-seq analysis. A 0 h sample was included as a control (mock) for RT-qPCR to validate RNA-seq results.

### Rice blast disease bioassay

Plant growth and Hrip1 induction were performed as previously described for RNA_Seq and qPCR. *M. oryzae* (KJ201) spore preparation and treatment were performed in accordance with Zhao et al. (2018) and Mondal et al. (2021) descriptions. In brief, *M. oryzae* (KJ201) spores were inoculated on oatmeal agar and incubated in darkness for 10 days at 30°C, then at 24°C to induce spore formation for 4 days. Spores on each plate were washed with 2000 μl ddH_2_O containing 0.25% tween-20 and diluted to 1 × 10^5^ spores ml^−1^. After 48 h of Hrip1 treatment, the rice seedlings were evenly sprayed with *M. oryzae* (KJ201) spores, covered with black polythene, and placed in the greenhouse at 25°C for 48 h in the dark. The 12 h photoperiod was restored afterward, and the temperature was set at 24°C in darkness, 28°C under light, and 85% relative humidity. Disease score analysis was performed after 7 days of continuous growth. The blast score was assigned based on the number and area covered by the lesions according to the 0–9 scale described by Li et al. (2021a,b).

### RNA extraction and cDNA construction

Total RNA was extracted using the Easy Pure Plant RNA Kit (Transgene Biotechnology Company) by following the manufacturer’s protocol. Total RNA (3 μg) was reverse transcribed into first-strand cDNA following the instructions and kit from Transcript all-in-one first-strand cDNA Synthesis SuperMix (Transgene Biotechnology Company).

#### Genome sequencing, quality assessment, and mapping

RNA extracted from 24 samples (triplicate samples per time point) of Hrip1-treated and buffer-treated seedlings were sequenced using the RICfoiR_BGISEQ-500 RNA_Seq platform (BGI Co., Ltd., Beijing, China), following the RICfoiR_BGISEQ-500 protocol. Quality control analysis was performed using the java program RNA-seQC to remove sequence adaptors, low-quality reads (bases with sequencing quality below 5), and reads with a high content of unknown bases greater than 10%. Clean reads were mapped to the reference genes and genome (Os-Nipponbare-Reference-IRGP-1.0) using Bowtie2 and HISAT.

#### DEGs, GO, and KEGG pathway analyses

DEGs screening between treatment and control groups was performed using the NOISeq package. The gene expression level of each sample was computed as log_2_ [foldchange], M, and absolute differences, D for all paired conditions, to build a noise distribution model.

$M^{i}=\log2\frac{X_{1}^{i}}{X_{1}^{i}}\;\mathrm{and}\;D^{i}=\left|X_{1}^{i}-X_{2}^{i}\right|\;$ $P_{A}=P\;\left(M_{A}\geq \left\{M\right\}\&\&D_{A}\geq \left\{D.\right\}\right)$

The mean expression levels of the control (Control_avg) and treatment (Treat_avg) groups were computed. The treatment and control averages were used to calculate the fold change, M_A_ and absolute difference D: (M_A_ = log2 ((Treat_avg)/ (Control_avg))) and D_A_ = |Congrol_avg-Treat_avg|. A gene is differentially expressed (DEG) if M_A_ and D_A_ diverge from the noise distribution model. Based on this, a default criterion was set to consider a gene as differentially expressed if the foldchange was ≥2 and the diverged probability was ≥0.8.

All DEGs were first mapped to GO terms^1^ by calculating the gene numbers for each term. A hypergeometric test was then performed to identify significantly enriched GO terms on the GO term finder platform.^2^

KEGG pathway enrichment analysis was performed using the public database for genes and genomes^3^ to enhance the overall understanding of the biological functions of the DEGs. KEGG analysis revealed DEGs with significant metabolic or signal transduction pathways than the genome background.

### RT-qPCR analysis

Verification of selected genes from the RNA_Seq data was performed on the QuantStudio 5 system using sequence-specific primers designed on primer premier 5 (Supplementary Table 1). The *OsActin1* (*Os03g0718100*) gene was used as the internal reference, and buffer-treated plants were taken at 0 h as control (mock). The 2× RealStar green fast mixture with ROX II kit (GenStar) was used to perform the analysis. A total of 20 μL qPCR mixture consisting of 1 μL cDNA, 0.5 μL each of forward primer and reverse primer, 8 μL RNA_free water, and 10 μL of 2× RealStar green fast mixture was prepared. The PCR condition was as follows: 95°C (2 min), 95°C (15 s), 60°C (30 s), and 72°C (30 s) for 40 cycles. The relative expression of genes was computed as described (Zhang et al., 2021) using the 2^−ΔΔCt^ method with three biological replicates.

### Phylogenetic analysis of disease-responsive genes

CDS sequences of DEGs were downloaded^4^ (Goodstein et al., 2012) to build neighbor-joining trees to investigate DEGs’ evolutionary and functional relatedness using the maximum composite likelihood method. The parameters used to construct the trees on MEGA7 were 1,000 bootstrap replications and the complete deletion of gaps and missing data (Kumar et al., 2016).

### Quantification of lignin

Lignin content was analyzed using the thioglycolic acid (TGA) method described by Bonawitz et al. (2014). Treated rice leaves were sampled at 0, 6, 12, 24, 48, 72, and 168 h into a 2 ml microcentrifuge tube containing a grinding ball (RETSCH 53680021, Al_2_O_3_ 10 mm). Samples were frozen in liquid nitrogen and pulverized into powder using a Retsch MM400 Laboratory Mixer Mill (20.745.0001, Germany). The grinding conditions were 4 min and 30 Hz. 100 mg of the homogenate was placed in a fresh 2 ml microcentrifuge tube, mixed with 10 volumes of 100% methanol, and extracted for 2 h at 80°C on a digital block heater. The extracts were centrifuged at 12,000 g, pellet was washed three times with 10 volumes of ddH_2_O. The pellet was then resuspended in a solution containing 750 μL ddH_2_O, 250 μL concentrated HCl, and 100 μl TGA and incubated at 80°C for 3 h. After centrifugation at 12,000 × *g*, the pellet was washed with 1 ml ddH_2_O, dissolved again in 1 ml of 1 M NaOH, and rocked in a shaking incubator at 28°C for 12 h. Samples were centrifuged at 12,000 × *g* for 10 min, and the supernatant was collected into a clean 2 ml microcentrifuge tube without disrupting the pellet. Concentrated HCl (200 μL) was added to the supernatant, vortexed to mix, and incubated at 4°C in a refrigerator. The lignin precipitate formed was collected by centrifugation at 12,000× *g* for 10 min, dissolved in 1 ml of 1 M NaOH, and absorbance was detected at 280 nm.

#### Transmission electron microscopy of cell wall morphology

TEM was performed as Mravec et al. (2017) described with little modifications. Leaf samples were quickly cut into approximately 3 mm^2^, then evacuated in 4 ml fixative buffer containing 2% glutaraldehyde (pH = 7.2) using a glass syringe. Samples were then completely submerged in 1 ml of the same buffer in a 1.5 ml pointed-end microcentrifuge tube and incubated at room temperature for 48 h. The sections were dehydrated in a graded series of ethanol solutions for 15 min each at 30, 50, 70, 80, 90, 95, and 100%. The specimens were transferred into freshly prepared resin: acetone (1:1) solution and left overnight at 35°C, then immersed in acetone for 60 min with six substitutions. Specimens were then polymerized by UV for 48 h. Ultra-thin sectioning (50–70 nm) was performed using an ultramicrotome. Finally, specimens were double-stained with uranium acetate and lead citrate and observed using the HITACHI H-7500 (Japan).

The ImageJ software was used to measure cell wall thickness at seven points per image.

### UPLC-MS/MS analysis of diterpenoids

Rice growth, elicitor treatment, and sample preparation were the same as those described for lignin content analysis. Leaf samples were obtained after 3 days of treatment and extracted with 20 volumes of 80% HPLC grade methanol. The extract was analyzed by UPLC-MS/MS in the multiple reaction monitoring (MRM) mode, as described by Kariya et al. (2020). The UPLC-MS/MS parameters included Colum: Acquity UPLC BEH C18 (2.1 × 50 mm, 1.7 μm). Waters: the flow rate was set at 0.2 ml/min, and the column temperature was at 40°C. The solvents used were 0.1% aqueous formic acid (A) and 0.10% formic acid in acetonitrile (B); a 42% (2 min) gradient, followed by a 42–47% B/(A + B; 8 min). Compounds were analyzed in the positive ion mode with nitrogen as the collision gas.

### Data analysis

Statistical analysis was performed using GraphPad Prism, version 9.0.0. The RT-qPCR data sets were subjected to a one-way analysis of variance using Dunnett’s multiple comparison tests. Lignin and cell wall thickness analyses were performed using the two-way ANOVA (Bonferroni’s multiple comparison tests). Violin plots of means for quantified diterpenoids were also performed on GraphPad prism 9.0.0. Chemical structures were drawn and analyzed using ChemDraw professional version 20.0.

## Results

### Gene amplification, protein expression, and purification

**Figure 1:**
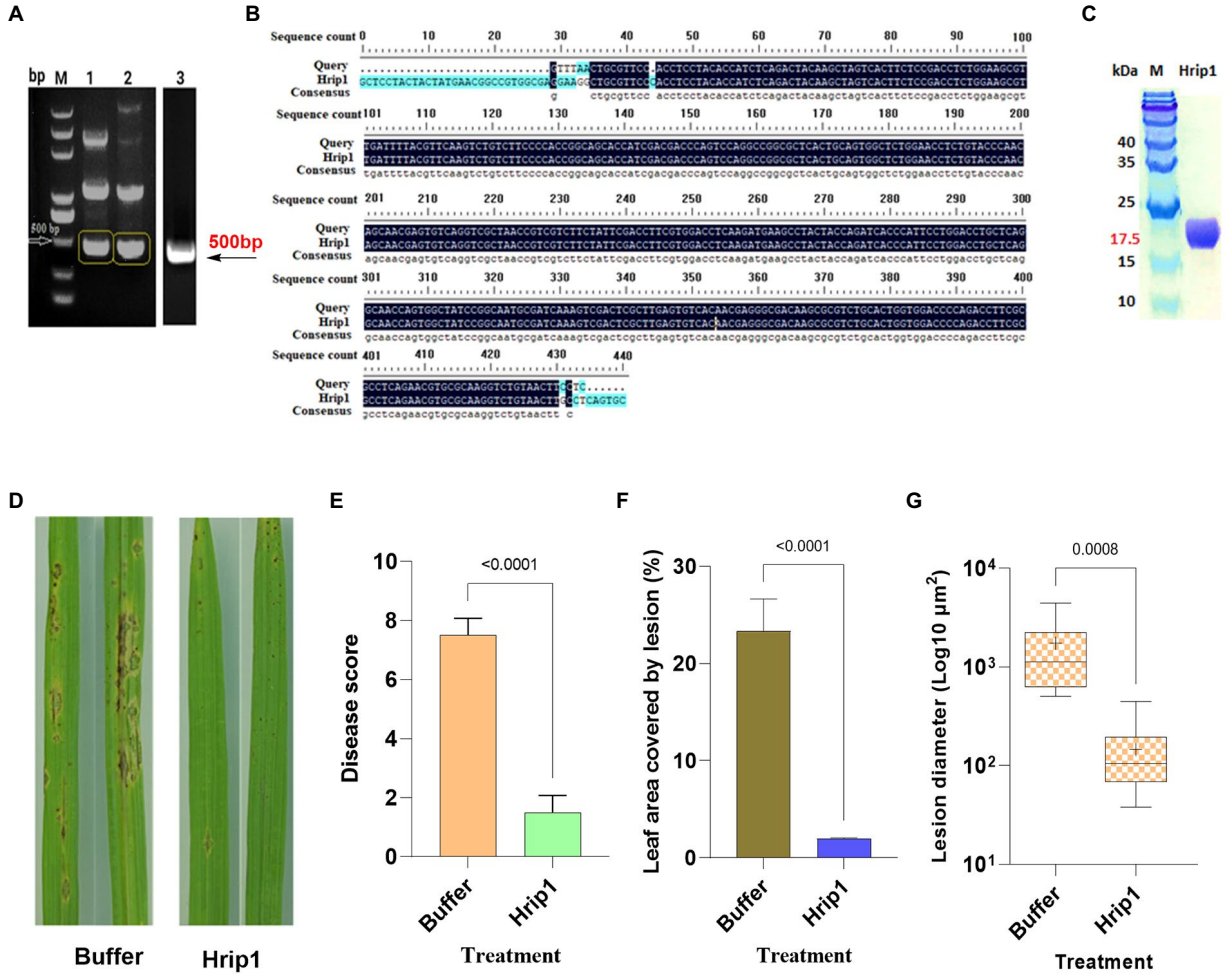
Protein expression and rice blast assay. **(A)** PCR amplified Hrip1 gene (500 bp). **(B)** Sequence alignment of Hrip1. **(C)** SDS-PAGE analysis of hrip1 protein (17 kDa). **(D)** Rice blast lesion features on leaves photographed after 7 days. **(E)** Rice blast scores (*n* = 2). **(F)** Leaf surface area covered by lesion (*n* = 2). **(G)** Mean diameter of lesions on the leaf (*n* = 11, “+” denote mean). For all graphs, error bars = mean ± sd, compared at a 95% confidence interval.

Hrip1 integrant was confirmed through PCR amplification and gel purification (Figure 1A). The purified PCR product was sequenced, and 92.02% sequence consensus with the reference gene was attained (Figure 1B). Hrip1 protein was then expressed and purified using the AKTA protein purification system, where the yeast elicitor was pooled down, and the Hrip1 secreted protein was collected. The purified Hrip1protein was further confirmed by detecting the correct protein band (17.5 kDa) on 12% SDS-PAGE (Figure 1C).

### Hrip1 induces rice immunity against *Magnaporthe oryzae*

Rice seedlings treated with 30 μm Hrip1 protein and 30 μM Tris–HCl, pH 8.0 as controls, were sprayed with *M. oryzae* (KJ201) spores after 48 h and were monitored for symptoms of blast lesions.

Sporadic needle-like lesions, less than 10^2^ μm in diameter, emerged in the control plants 48 h after treatment. However, no symptoms were observed in Hrip1-treated plants. The size and number of lesions in the control plants increased with sporulation commencing after 96 h, whereas only a few tiny separate lesions appeared around the tips of the Hrip1-treated leaf (Figure 1D). The disease score determined 7 days after inoculation showed that Hrip1-treated plants developed significant resistance to blast infection with a mean score of 1.5. In contrast, the control plants were significantly susceptible, with a mean score of 7.5 (Figure 1E). The dense lesions on the control plants recorded an average diameter of 10^3^ μm, covering about 24% of the leaf’s total surface area. Moreover, the separate lesion developed on Hrip1-treated plants has an average size of 10^2^ μm^2^ and covers less than 2% of the total surface area of the leaf, suggesting the involvement of Hrip1 in immunity induction (Figures 1F,G). Following the bioassay results, transcriptome and metabolic profiling analyses were conducted to investigate the intricate role of Hrip1 in immunity induction.

### Genome sequencing, assembly, and annotation

Transcriptome analysis was performed to investigate the transcriptome changes activated by Hrip1 treatment. The sequencing results generated 23,957,585 average raw reads and 23,936,075 average clean reads (Supplementary Table 2). An average of 22,916,996 reads was obtained when clean reads were mapped to the reference genome using HISAT (Kim et al., 2015; Supplementary Table 3) and 21,089,976 average clean reads achieved when mapped to reference genes (Supplementary Table 4). Quality control checks conducted on the sequencing data to ascertain their dependability affirmed that the sample sequencing data eclipsed the clean read threshold of ≥90% (91.90–97.9%). Furthermore, the uniquely mapped gene threshold ratio of 80% and the genome mapping threshold ratio of 50% both passed the quality control test with values ranging from (86.26–90.32%) and (93.34–98.27%), respectively (Supplementary Table 5).

#### Statistics of differentially expressed genes

DEGs were analyzed using the NOISeq tool, and the standard for selection was set at a fold change ≥2 and divergence probability ≥0.8. The transcriptome analysis yielded 471, 409, 491, and 1,088 upregulated and 6, 31, 1, and 9 downregulated genes at 6, 12, 24, and 48 h, respectively (Supplementary Figure 1A). Scatter (Supplementary Figure 1B) and volcano (Supplementary Figure 1C) plots for each pairwise comparison were generated based on the DEGs threshold to illustrate the distribution of upregulated, downregulated, and non-responsive genes to Hrip1 treatment. Generally, the sequencing statistics showed that Hrip1 treatment yielded more upregulated genes than downregulated ones. Comparing DEGs on Venn diagrams revealed unique and common DEGs among sampling points. In all triplicate point comparisons, commonly expressed genes were more highly expressed in all Hrip1-treated groups than in the control group (Supplementary Figure 1D).

#### Gene ontologies and KEGG pathway analysis

DEGs were assigned to three ontology terms: biological process (BP), cellular component (CC), and molecular function (MF). GO enrichment analysis identifies enriched ontologies in the DEGs compared with the genome background (Supplementary Figure 2). Metabolic and cellular processes, localization, and responses to stimuli were the most enriched BP terms in all the pairwise comparisons. The highly enriched common GO terms under MF include binding, catalytic, and antioxidant activity. However, structural molecular activity was enriched only after 48 h. Cell, cell parts, organelles, and macromolecular complexes were the most enriched CP terms at all-time points.

The KEGG pathway-based analysis was performed to aid further understanding of the biochemical roles of DEGs, such as signal transduction or metabolic pathways (Dunlap et al., 2013). Three disease-responsive pathways (Supplementary Figure 3) were identified among the top 20 KEGG annotations: phenylpropanoids/lignin, diterpenes, and benzoxazinoids (Supplementary Figure 4).

### Hrip1 induces transcriptional reprograming and defense modulation

Studies have shown that plants rely on single or multiple metabolites for defense and environmental cues (Weng et al., 2021). The accumulation of plant metabolites is controlled at the transcriptional level by a combinatorial interplay of DNA and transcriptional factor-related activities (Colinas and Goossens, 2018). Genes encoding metabolic synthesis play crucial roles in defense trade-offs, and exogenous elicitor treatments can activate their expression. Hrip1 treatment activated genes encoding three defense-responsive pathways for enhanced immunity.

#### Hrip1 activates cell wall biosynthetic genes and MYB transcriptional factors

**Figure 2:**
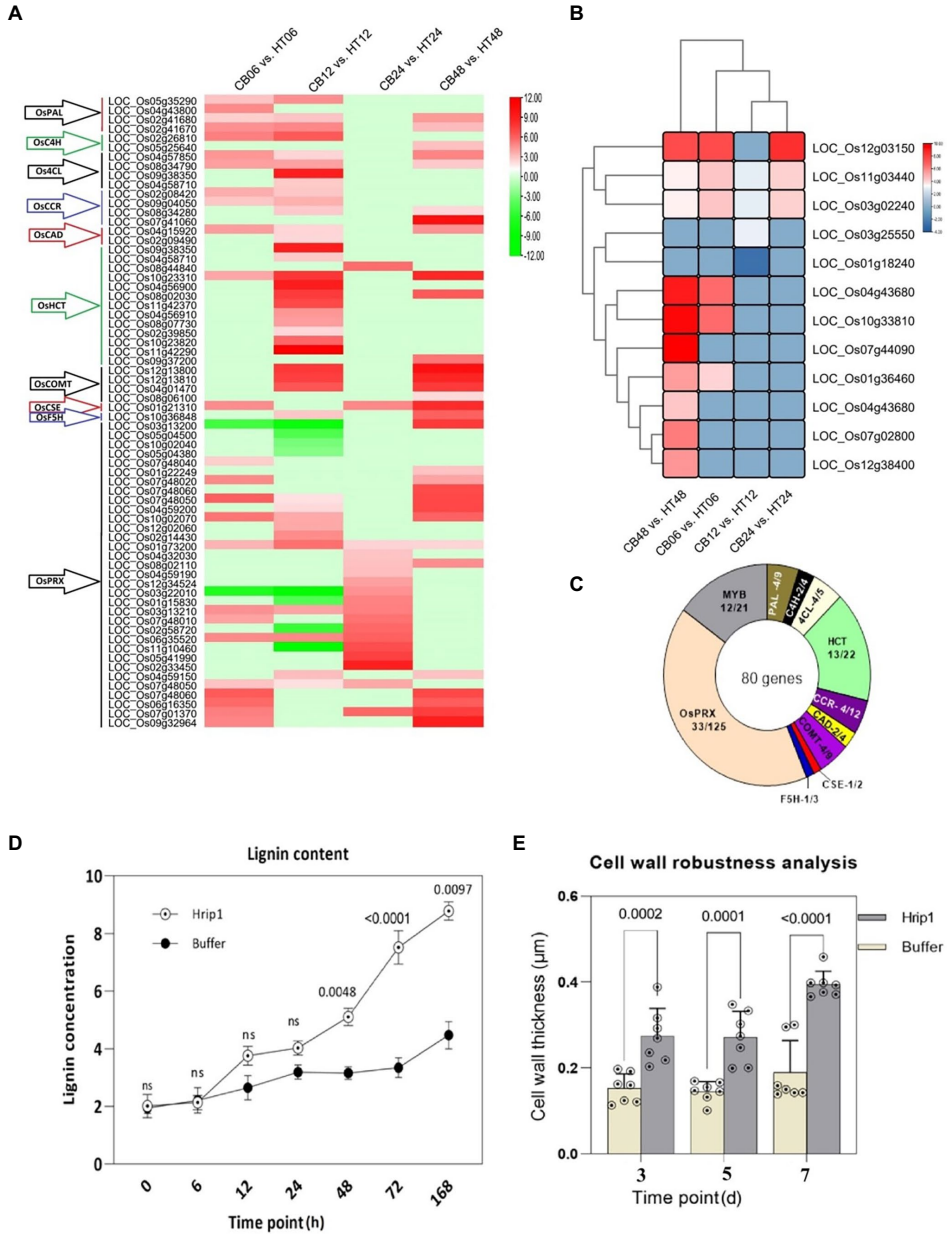
Lignin biosynthetic genes and transcriptional factors activated after Hrip1 treatment. **(A)** Expression profile of Hrip1-treated lignin biosynthetic genes, heat maps represent log2 (fold) values from the transcriptome results. Each time point represents a pairwise comparison between treatment and control. The gene families are indicated with arrows to the left: Phenylalanine ammonia-lyase (PAL), Cinnamate-4-hydroxylase (C4H), 4-hydroxycinnamoyl-CoA ligase (4CL), Hydroxycinnamoyl transferase (HCT), Cinnamoyl CoA reductase (CCR), Cinnamoyl alcohol dehydrogenase (CAD), Ferulate 5-hydroxylase (F5H), Caffeic acid 3-*O*-methyltransferase (C*O*MT), Caffeoyl shikimate esterase (CSE), and Peroxidase (PRX). **(B)** Neighbor-joining phylogeny of induced lignin biosynthetic genes. **(C)** Clustering and expression pattern of MYB transcription factor. Statistics of all Hrip1-treated genes associated with lignin metabolism and polymerization compared with the rice genome. **(D)** Lignin content analysis (*n* = 2). **(E)** Cell wall thickness analysis (*n* = 7).

Hrip1 induced several cell wall biosynthesis genes. The functions of these genes span the entire monolignol biosynthesis and polymerization pathway, including the MYB TF (Figure 2). A total of 35 DEGs encoding monolignol biosynthesis and 33 DEGs involved in monolignol polymerization (peroxidase) were identified (Figure 2A; Supplementary Table 6). A neighbor-joining tree constructed using CDS sequences on Mega7 showed these genes are functionally related (Supplementary Figure 5A). The phylogeny analysis also showed that PAL, COMT, and HCT share ancestral roots with PRX while CAD, CCR, 4CL, and F5H belong to another ancestral group.

A total of 12 MYB TFs out of the 21 known members of the rice genome were identified. MYB TFs possess rich adenosine and cytosine (AC) motifs that activate lignin biosynthesis genes (Geng et al., 2019). Their high expression showed a positive indication of enhanced lignin accumulation. Moreover, the expression levels of these genes significantly increased after 48 h of treatment, suggesting that lignin accumulation and cell wall-wall-mediated immunity could exceed 48 h before maximum immunity is attained (Figure 2B). The clustering plan of the MYB TF genes showed that they are related in function and may have contributed to lignin accumulation. Collectively, 80 genes were involved in lignin biosynthesis following Hrip1 treatment. Compared to the rice genome, Hrip1 induced 52.23% monolignol biosynthetic genes, 26.4% monolignol polymerization genes (PRX), and 57.14% MYB FTs (Figure 2C). The combinatorial activities of these genes could enhance cell wall robustness for rice defense against invasions.

#### Activated lignin genes and MYB TFs enhance lignin accumulation and cell wall thickness

We also demonstrated that the enrichment of cell wall biosynthesis genes, and MYB TFs, resulted in lignin accumulation. Following the high expression profile of these genes after 48 h, the sampling time point of treated seeding was expanded to include 72 and 168 h after treatment. The results showed that lignin content in Hrip1-treated seedlings gradually increased over time. Between 12 h and 24 h, Hrip1-treated plants slightly accumulated more lignin than the control plants. However, lignin content increased by 68.45% in the treated plants after 48 h onwards (Figure 2D). The differential lignin accumulation between the treated plants suggests the involvement of upregulated lignin synthetic genes and their MYB transcriptional activators.

**Figure 3:**
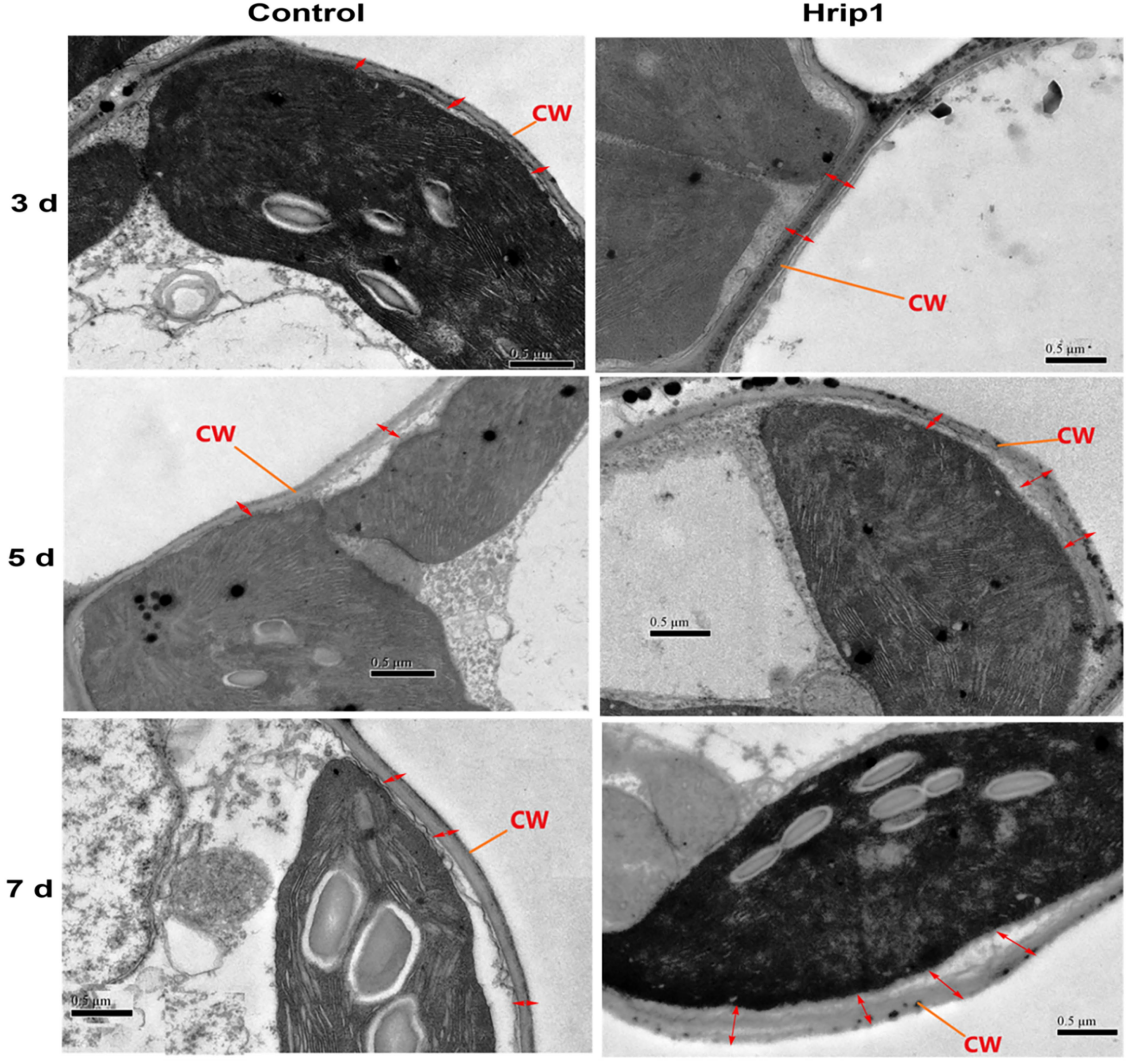
Transmission electron microscopy study of cell morphology. Samples were observed and photographed at 25,000× magnifications. Photos on the left represent the control group, and on the right are Hrip1-treated.

Cell wall morphology analysis was performed to confirm the role of lignin accumulation in cell wall thickening. Treated leave samples were obtained at 3, 5, and 7 days, following high lignin accumulation after 48 h. Cell wall thickness in Hrip1-treated leaves was enhanced by over 100% compared to the buffer-treated leaves (Figure 2E). Similar to lignin accumulation, cell wall thickness increases with time (Figure 3). The gradual accumulation of cell wall polymer in building a robust cell wall shows that Hrip1-induced immunity conference is systematic and lasts for several days. The coexpression of lignin and MYB genes further suggests that Hrip1-induced immunity might have relied on MYB switches to activate lignin biosynthesis for cell wall-mediated immunity.

#### Hrip1 activates diterpenes synthases

**Figure 4:**
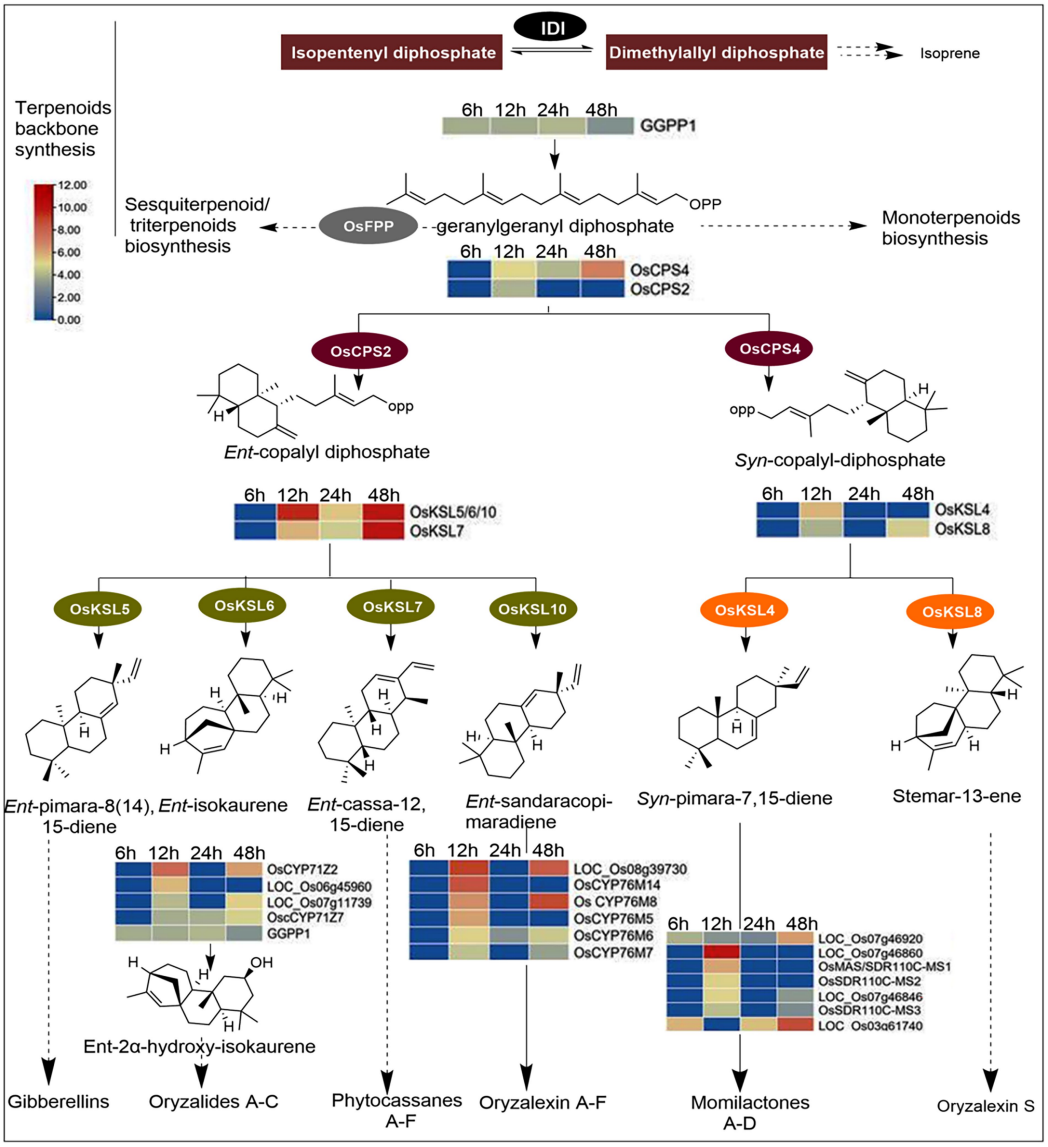
Diterpenoids biosynthesis pathway illustrating the functions of Hrip1-activated genes. Heat maps were constructed from log2fold values of DEGs. Genes encoding steps with dash arrows were non-responsive to Hrip1 treatment. Diphosphate isomerase (IDI).

Rice plants accumulate labdane-related diterpenoids under stressful conditions, such as pathogen attacks. The biosynthesis of these phytoalexins is controlled by specialized genes called diterpene synthases, and their expression has a greater influence on the amount of phytoalexin elicited by the plant. Hrip1 activated 24 genes encoding diterpene synthases, including the geranylgeranyl diphosphate precursor (OsGGPPS1). Class II diterpene synthases, *ent*- and *syn*-copalyl diphosphate synthases (OsCPS2 and OsCPS4) were also responsive to Hrip1 treatment. In addition, six class I diterpene synthases (Kaurene), termed as KS or KSL in most plants, were upregulated. Four are paralogs of OsCPS2 (OsKLS5, OsKLS6, OsKLS7, and OsKLS10), which encode gibberellins, oryzalides phytocassanes, and oryzalexins production. The remaining two (OsKLS4 and OsKLS8) are paralogs of OsCPS4 for synthesizing the carbon skeletons of momilactones and oryzalexin S. Additionally, two cytochrome P450 subfamilies, CYP71Z (4 genes), CYP76M (6 genes), and seven momilactone synthases, were highly expressed after Hrip1 treatment. These genes catalyze the final conversion of oryzalides, oryzalexins, and momilactones from their terminal precursors. Based on the KEGG annotation and the expression patterns of these genes, the stages in the diterpene pathway they encode are illustrated using log2 (fold) values (Figure 4).

Schmelz et al. (2014) identified 20 genes involved in diterpenoids biosynthesis. However, Hrip1 treatment induced the upregulation of 24 diterpenoid synthases. A neighbor-joining phylogeny constructed from CDS sequences showed these genes are ancestrally related. From the phylogenetic tree, CPS (II) and the KLS (I) genes are descendants of a common ancestor. The same observation was made for CYP70M (VI) and CYP71Z (V) genes, sharing an ancestral root with GGPP precursor (IV). The clustering plan of these genes on the phylogeny showed they are functionally related (Supplementary Figure 5B).

#### Genes encoding the stepwise conversion of indole-3-glycerol phosphate to DIBOA, TRIBOA-glycosides, and DIMBOA-glycosides are responsive to Hrip1 treatment

**Figure 5:**
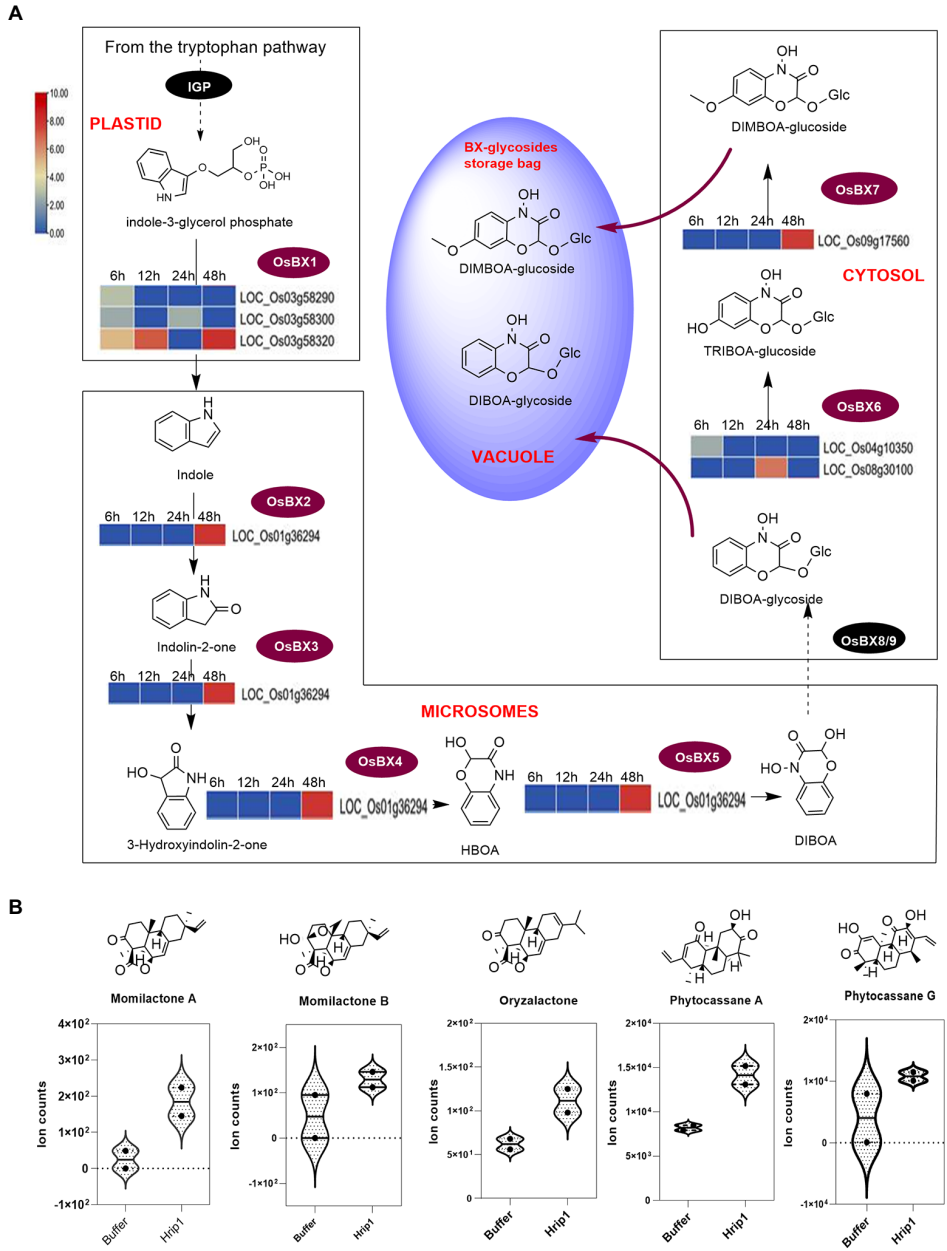
DEGs encoding BX biosynthesis and phytoalexin diterpene contents in rice seedlings. **(A)** BX biosynthesis pathway showing the steps each gene encodes. Reaction steps are classified according to their sites of occurrence (organelles). BX-glycosides are finally deposited in the vacuole and elicited upon biotic stress perception. **(B)** UPLC-MS/MS analysis of diterpenoids (*n* = 2).

Following Hrip1 treatment of rice seedlings and sequencing, seven upregulated genes in the BXs pathway were identified. Three genes encode indole-3-glycerol-phosphate lyase (BX1) to convert indole-3-glycerol phosphate to indole, a committed step in BXs biosynthesis (Tzin et al., 2017). A cytochrome P450 superfamily gene (*LOC_Os01g36294*) was identified to overlap in function as BX2, BX3, BX4, and BX5 to produce DIBOA. Two genes encoding 2, 4-dihydroxy-1, 4-benzoxazin-3-one-glucoside deoxygenate (BX6) for synthesizing TRIBOA-glycoside were upregulated. Also, a 2, 4, 7-trihydroxy-1, 4-benzoxazine-3-one-glucoside 7-*O*-methyltransferase (BX7) gene which encodes the conversion of TRIBOA-glycoside to DIMBOA-glycoside for storage in the vacuoles was identified (Figure 5). The clustering pattern of these genes shows they are functionally related (Supplementary Figure 5C).

#### Activated diterpene synthases and BX genes induce phytoalexin accumulation

The effect of Hrip1-treated gene activation on diterpene accumulation was verified by metabolic analysis using the UPLC-MS/MS technique. Hrip1 treatment enhanced the production of phytocassane A and momilactones A and B compared to the control plants. Kariya et al. (2020) recently isolated an isomer of momilactone A, named oryzalactone, and a di-dehydrated phytocassanes A, named phytocassanes G. These diterpenoids inhibited *M. oryzae* conidia by 86 and 45%. Phytocassane G and oryzalactone were detected in both Hrip1-treated and control plants. However, Hrip1-treated leaves accumulated more of these metabolites than the control seedlings (Figure 5B).

The defensive trademarks of benzoxazinoids are well established and reported. They are involved in both aerial and below-ground defense against pests and diseases. Though we could not verify the amounts of BX accumulation due to the absence of authentic standards, reports on wheat and maize have demonstrated that transcript abundance results in their high accumulation and defense against diseases and pests (Duan et al., 2021; Sue et al., 2021).

### Robustness test of the transcriptome analysis

**Figure 6:**
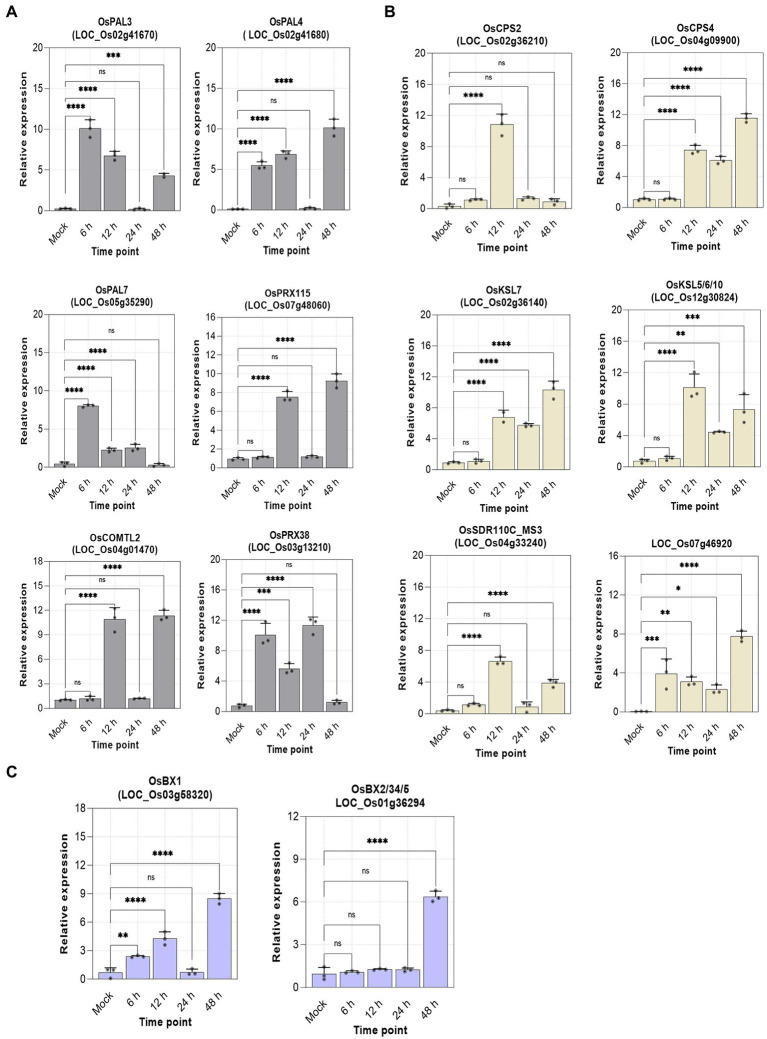
Relative expression of selected genes. *n* = 3, error bars = mean ± sd, *p*-value >0.01(*), *p*-value >0.001(**), *p*-value >0.0001(***), and *p*-value >0.00001(****) at 95% confidence interval. **(A)** Lignin genes; **(B)** diterpenoids genes; **(C)** benzoxazinoids genes.

The robustness of the transcriptional data was confirmed by RT-qPCR validation of selected genes from each metabolic pathway. The relative expression of lignin (6), diterpenoids (6), and benzoxazinoids (2) biosynthetic genes collaborates with the transcriptome data (Figure 6). This suggests the crucial involvement of Hrip1 in activating defense pathways to confer immunity against rice blast fungi.

## Discussion

Plants develop intricate mechanisms to withstand antagonistic pathogens. Their immune systems recruit secondary metabolites whose accumulation is controlled at the transcriptional level for defense and environmental cues (Li et al., 2021a,b). Transcriptional profiling techniques have been used to unearth complex metabolic machinery triggered by elicitor treatment and have therefore become a stopgap tool in functional genomics. RNA sequencing has established itself as a leading whole-genome transcript quantification technology that produces reliable results (Lachmann et al., 2018). Here, we present the transcript changes induced by Hrip1 in *O. sativa* immunity against rice blast fungi. Three defense metabolic pathways: (a) phenylpropanoids/lignin, (b) diterpene, and (c) BXs pathways, are induced by Hrip1 treatment and contributed to the physical and chemical resistance against the rice blast fungus.

Hrip1 defense activation is similar to Mohrip1 and Mohrip2. Lv et al. (2016) reported that Mohrip1 promotes growth and defense against rice blast fungi by activating the expression of genes encoding SA, gibberellin, and diterpenoid biosynthesis, in addition to pathogenesis-related proteins such as NAC. Moreover, MoHrip1 and MoHrip2 transgenic rice plants enhanced immunity against rice blast disease *via* SA induction (Wang et al., 2017). In contrast to Mohrip1 and Mohrip2, which induce fewer genes encoding diterpenes (8), NAC (2), and PAL (1), Hrip1 activated 68 genes encoding all steps in the lignin pathway, 12 MYB TFs, and 24 diterpene synthases. Moreover, unlike Mohrip1 and Mohrip2, Hrip1 activated the expression of BXs genes (7).

Lignin is a hydrophobic aromatic polymer abundant in plant secondary cell walls and is synthesized *via* the phenylpropanoid pathway. It is partly responsible for bundling cellular components to enhance CWI rigidity for PTI induction. A lignified cell wall also confers physical barriers to pathogens such as fungal haustoria and restricts trans-cellular infections (Cui et al., 2018; Vermaas et al., 2019; Wan et al., 2021). Lignin also modulates insect attacks, as He et al. (2020) demonstrated that brown planthopper (BPH) lost its piercing efficiency against rice hosts after lignin and SA accumulation were activated by R2R3 MYB TF induced expression of *OsPAL* genes. Li et al. (2021a,b) revealed the role of *OsPAL* and other phenylpropanoid genes in lignin, SA, melatonin, and flavonoids’ defense against leaf folder herbivores and BPH.

Hrip1 mediates cell wall immunity by inducing the accumulation of 68.45% lignin in rice leaves, resulting in 100% cell wall rigidity. Our findings are consistent with several reports demonstrating that cell wall-associated genes in isolation or combination encode lignin accumulation and defense modulation (Govender et al., 2017; Yuan et al., 2019; Li et al., 2020a,b; Chen et al., 2021a,b). For example, *OsPAL* genes induce immunity against *M. oryzae*, *Rhizoctonia solani*, and *Xanthomonas oryzae* pv *oryzae* (*Xoo*; Duan et al., 2014; Tonnessen et al., 2015). C4H encodes *p*-coumaric acid hydroxylation from cinnamic acid, which drives lignification and biosynthesis of other essential defense metabolites (Li et al., 2020a,b; Wang et al., 2020). The soybean *C4H1* gene has been linked to defensive lignification against *Phytophthora sojae*. Furthermore, Agrobacterium-mediated transformation of the CAD2 gene from *Pyrus pyrifolia* (pear) into tomato plants significantly increased lignin accumulation in the leaves, stems, and fruits (Li et al., 2019). The CAD gene family encodes the NADPH-dependent reduction of hydroxy-cinnamaldehydes to monolignol alcohols, leading to physical reinforcement of cell walls (Park et al., 2018).

Transcriptional factors such as *OsNAC5* mediate lignin biosynthesis by activating *OsCCR10* (Bang et al., 2021). Also, adenosine and cytosine enrichment of DNA motifs promotes lignin production by binding to MYB TFs. *MYB46* and its *MYB83* homolog increase phenylpropanoid and lignin biosynthesis (Chen et al., 2021a,b). Moreover, *AtMYB15* and *OsMYB30* were reported to regulate PAL, C4H, 4CL, HCT, C3H, COMT, and CAD to enhance lignin accumulation and defense against *Pst* DC3000 (*AvrRpm1*) and BPH, respectively (He et al., 2020; Kim et al., 2020). Chezem et al. (2017) also showed that *AtMYB15* induced lignification and basal immunity in Arabidopsis by binding to cell wall-responsive apparatus containing AC components. A total of 12 MYB genes are linked to Hrip1 induction. Their complementary roles in lignin accumulation might have influenced the cell wall thickness, especially *OsMYB61*, which enhances 53% culm tissue lignin and cell wall enrichment when overexpressed in rice protoplast (Zhao et al., 2019).

There is no consensus regarding the transport of lignin monomers into the apoplast. Some schools of thought suggest active transport mechanisms are involved in moving glycosylated lignin monomers mediated by ABC transporters. Other studies have linked monomeric transport *via* passive diffusion (Miao and Liu, 2010; Tsuyama et al., 2013; Shimada et al., 2021). Notwithstanding these contrasting views, monolignol transport is crucial for lignification. Peroxidases (hydrogen peroxide) and laccases (molecular oxygen) genes encode lignin polymerization for extracellular support, nutrient transport, and defense (Xie et al., 2020). Hrip1 activated 33 PRX genes that contributed to cell wall-mediated immunity. Consistent with our results, Meng et al. (2021) identified *DcPrx30*, *DcPrx32*, and *DcPrx62* as lignification genes in carrot. In *Arabidopsis*, PRX33 and PRX34 activate PTI. Confirmatory knockdown of these genes compromised H_2_O_2_ content in response to PAMP treatment and PAMP-induced protein expression (O'Brien et al., 2012)

Plant defense is also activated by phytochemical elicitation (Nasir et al., 2018). Plants inductively or constitutively accumulate defense metabolites from biochemical pathways to enhance PTI (Mujiono et al., 2021). Diterpene accumulation is associated with *M. oryzae* and *Xoo* infections (Zhan et al., 2020). Momilactone (A and B), phytocassane (A and G), and oryzalactone levels were higher in Hrip1-treated plants than in the control groups. The biosynthesis of these metabolites is encoded by well-characterized synthases initiated by a C-20 prenyl-substrate, the GGPP precursor. Two gene clusters on chromosomes Os02g-*ent*- and Os04g *syn*-CPS and kaurene synthase have been reported to play crucial roles in the downstream biosynthesis of diterpenoids (Jia et al., 2019). The results reported here showed that *ent*-CPS2 and *syn*-CPS4 genes and their KSL paralogs were activated by Hrip1. *OsCPC2* and *OsCPS4* encode kaurenes. The *OsKSL* paralogs also encode the formation of the carbon skeletons of labdane-related phytoalexins. These two steps are essential for the downstream biosynthesis of phytoalexins (Miyamoto et al., 2016). *OsCPC2* and *OsKLS7* enrichment might have accounted for the increased phytocassane A and G levels in Hrip1-treated plants compared to control plants.

Furthermore, short-chain dehydrogenases/reductases (SDRs) are a large family of NAD- or NADP-dependent genes that oxidize 3-hydroxy-syn-pimaradien-19, 6-olide to a typical momilactone A carbon-3 (C3) keto group. Therefore, SDR genes are classified as momilactone synthases (Kitaoka et al., 2016; Miyamoto et al., 2016; Mao et al., 2020). Hrip1 activation of SDR genes (*OsSDR11C-MS1*, *OsSDR11C-MS2*, and *OsSDR11C-MS13*) might have accounted for the enhanced levels of momilactone A and B. Also, cytochrome P450 plays a crucial role in *ent*-sandaracopimaradiene hydroxylation to form oryzalexin diterpenoids. Wu et al. (2013) reported that CYP76M6 and CYP76M8 were involved in a non-redundant reaction with 3α-hydroxy-*ent*-sandaracopimaradiene to form oryzalexin. Hrip1 induces cytochrome CYP76M sub-family members, which catalyze oryzalexin formation. Hrip1 also enhanced the expression of four cytochrome P450 sub-family genes (CYP71Z) that participate in oryzalide biosynthesis. For example, OsCYP71Z2 is reported to induce resistance to *Xoo* infection (Li et al., 2013).

BXs are encoded by genes serially named BX1, BX2, and BX3, depending on the reaction step they catalyze. The role of BX1 in converting indole-3-glycerolphosphate to indole is a significant step in BXs biosynthesis (Tzin et al., 2017). Hrip1 activated seven BXs genes in the rice genome. Although we could not quantify these metabolites due to the absence of authentic standards, reports on wheat and maize showed that transcript abundance of BX genes results in high BX glycoside accumulation (Duan et al., 2021; Sue et al., 2021).

Our findings show that Hrip1 adopts a dual-barrier approach in defense modulation. (1) Hrip1 induced the upregulation of lignin and MYB TFs to confer cell wall-mediated immunity. (2) Hrip1 also increased the expression of genes encoding diterpenoids for phytoalexin accumulation. The enrichment of BXs genes might have contributed to rice immunity against *M. oryzae*. Other defense-significant metabolites such as lignans, coumarins, stilbenes, and caffeic acid might have also been induced to accumulate since their elicitation partly depends on lignin synthetic genes. This study provides novel insights into the mechanisms underlying Hrip1-treated immunity in plants.

## Data availability statement

The datasets presented in this study can be found in online repositories. The names of the repository/repositories and accession number(s) can be found at: https://www.ncbi.nlm.nih.gov/geo/query/acc.cgi?acc=GSE211516, with accession: GSE211516.

## Author contributions

HZ, VN, and JY: conceptualization. VN, JY, BL, LZ, and SL: protein purification. VN, LZ, JY, SL, ZF, and TY: transcriptome analysis and RT_qPCR. VN, JR, JD, and TY: metabolic analysis. VN, TY, and VN: plant growth and elicitor treatment. HZ: supervision of experimental procedures and data analysis. VN: first draft of the manuscript. HZ, XY, GL, and JR: revision of the manuscript. All authors contributed to the article and approved the submitted version.

## Funding

This research was supported by the National Key Research and Development Program of China, grant number 2017YFD0200900.

## Conflict of interest

The authors declare that the research was conducted in the absence of any commercial or financial relationships that could be construed as a potential conflict of interest.The reviewer J-YC declared a shared affiliation with the authors to the handling editor at the time of review.

## Publisher’s note

All claims expressed in this article are solely those of the authors and do not necessarily represent those of their affiliated organizations, or those of the publisher, the editors and the reviewers. Any product that may be evaluated in this article, or claim that may be made by its manufacturer, is not guaranteed or endorsed by the publisher.

## Footnotes

^1^http://www.geneontology.org/ (Accessed August 14, 2022).

^2^http://www.yeastgenome.org/help/analyze/goterm-finder (Accessed August 14, 2022).

^3^https://www.kegg.jp/kegg/kegg1.html (Accessed August 14, 2022).

^4^https://phytozome-next.jgi.doe.gov/ (Accessed August 14, 2022).
