# Supplementary material for: Hrip1 mediates rice cell wall fortification and phytoalexins elicitation to confer immunity against Magnaporthe oryzae
Source: Front Plant Sci. 2022 Sep 23;13:980821. doi: 10.3389/fpls.2022.980821 (PMC9546723; doi:10.3389/fpls.2022.980821)
Supplement: Supplementary file 1 [file Data_Sheet_1.docx]

**Hrip1 mediates cell wall fortification and phytoalexins elicitation to confer immunity against *Magnaporthe oryzae***

Vincent Ninkuu, Jianpei Yan, Lin Zhang, Zhenchao Fu, Tengfeng Yang, Shupeng Li, Beibei Li, Jiaqi Duan, Jie Ren, Guangyue Li, Xiufen Yang, Hongmei Zeng**^†^**^[[1]](#footnote-1)^

*State Key Laboratory for Biology of Plant Diseases and Insect Pests, Institute of Plant Protection, Chinese Academy of Agricultural Sciences, Beijing, 100193, China.* [zenghongmei@caas.cn](mailto:zenghongmei@caas.cn) (H. Z, ORCID ID: 0000-0001-7315-245X).

**
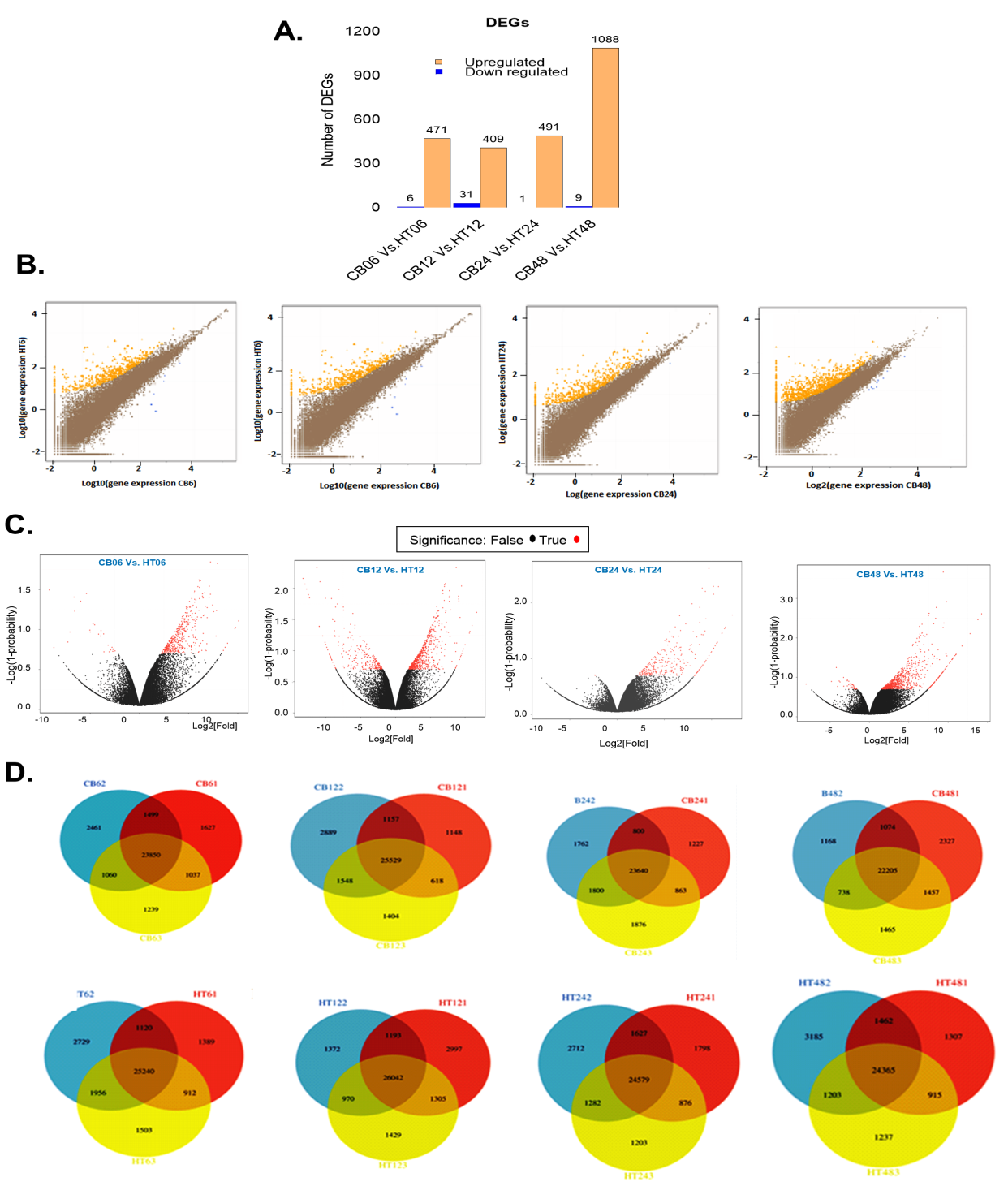
**

**Supplemental Figure 1:** Statistics of expressed genes

**(A) Differentially expresses gene under pairwise comparison (B) Scatter plots of all the expressed genes in each pairwise comparison; t**he X and Y axes show the log10 gene expression values for treatment (HT) and control (CB), respectively. Blue plots represent down-regulated genes, orange plots represent upregulated

**(a)**

**(c)**

genes, and brown plots represent non-responsive genes. If a gene is only expressed in one sample, its expression value in another sample is replaced by the minimum value of all expressed genes in the control and treatment samples. The screening threshold was set at probability ≥ 0.8 and (Log2(Y/X)) ≥ 1 (C) Volcano graphs of all the expressed genes in each pairwise comparison. The log-transformed threshold values are represented on the X and Y axes. Red and black dots represent significant and non-significant DEGs, respectively (C) Cluster analysis of DEGs in pairwise comparisons between treatment and control samples. The heatmap was built from Log2 (fold change) values of genes that were only differentially expressed in all pairwise comparisons (D) Venn diagrams of common DEGs among sampling points: Each diagram represents the triplicate samples obtained at the four different time points. Venn diagrams figures are proportional for two samples and not all three samples in each case. CB = control samples, 6, 12, 24, and 48 are the time points of sampling, and the last digit represents the triplicate sample number (1, 2, 3)

**Supplemental Figure 2:** GO terms enriched under each pairwise comparison between the Hrip1 treatment (HT) and control (CB) groups **(A)** CB6 vs. HT6 **(B)** CB12 vs. HT12 **(C)** CB624 vs. HT24 **(D)** CB48 vs. HT48, 6, 12, 24, and 48 are the sampling time points.

**
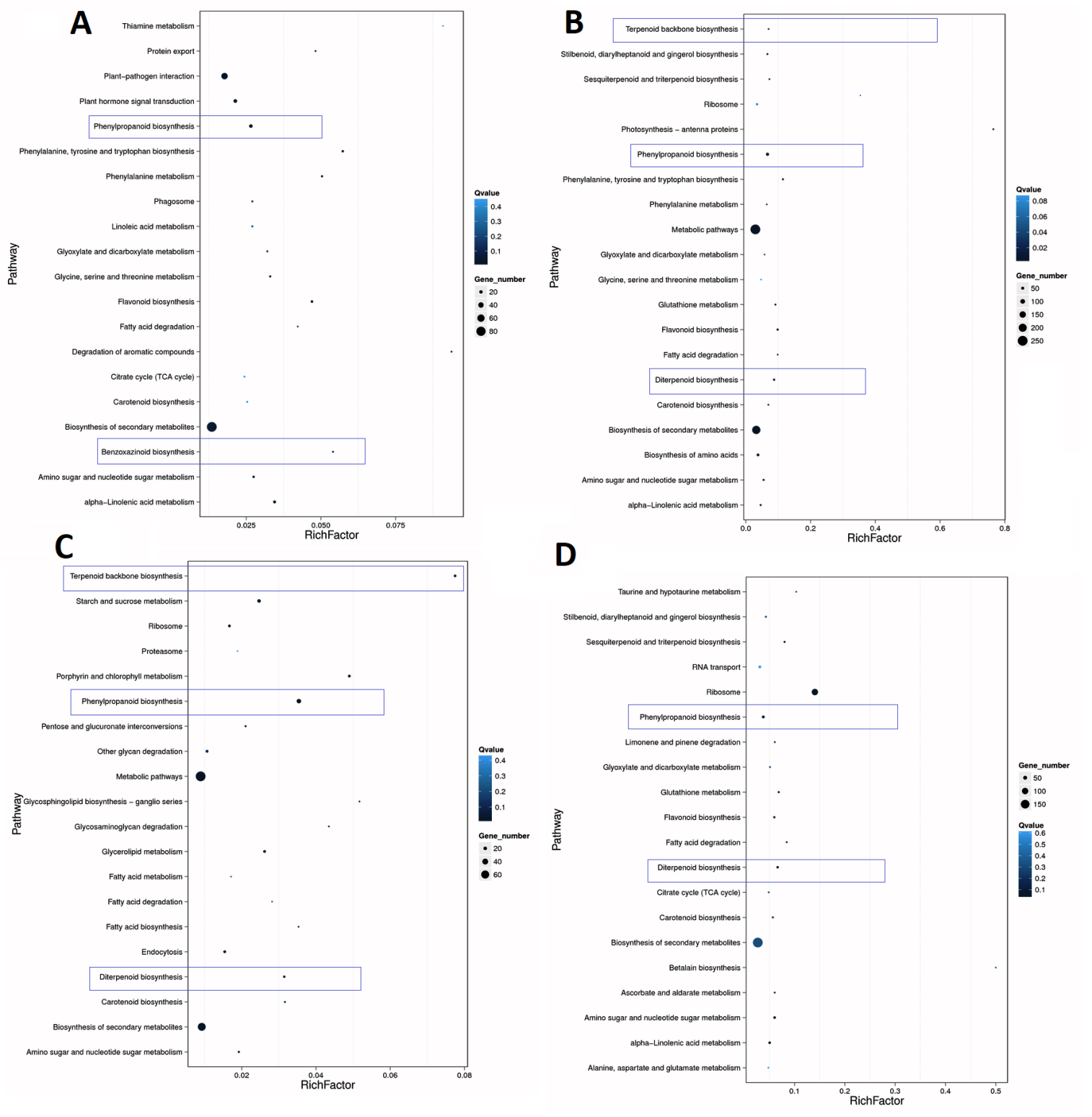
**

**Supplemental Figure 3: KEGG pathway enrichment of DEGs in each pairwise comparison:** RichFactor is the ratio of differentially expressed gene numbers annotated in the pathway term to all gene numbers annotated in this pathway term. Greater richFator means greater intensiveness. Q-values are corrected p-values ranging from 0~1, and a lower Q-value means greater intensiveness. The top 20 enriched pathway terms are displayed according to the pairwise comparison between treatment and control: (A) HT06 vs. CB06, (B) HT12 vs. CB12 (C), HT24 vs. CB24 (D).HT48 vs. CB48


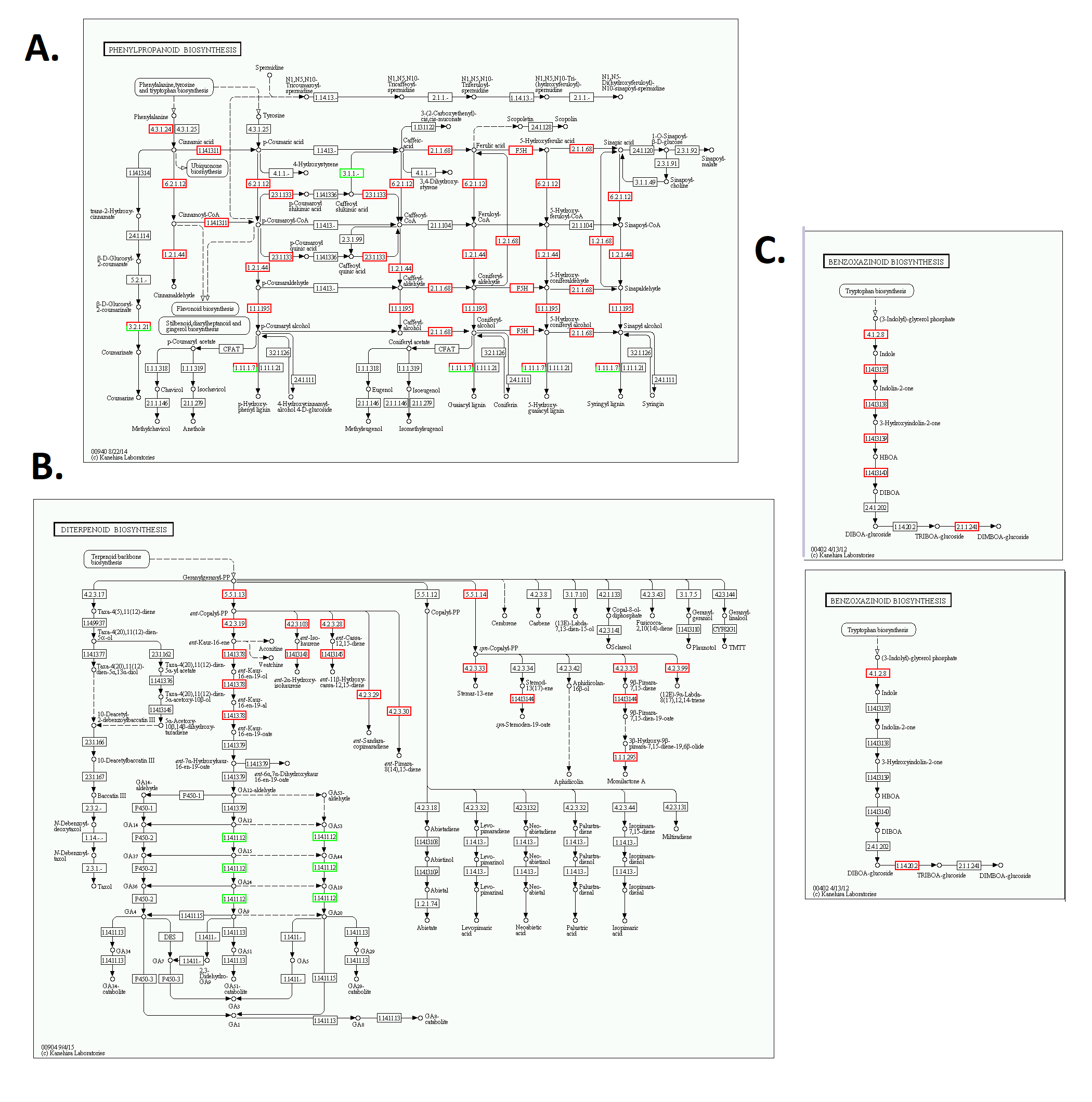


**Supplemental Figure 4:** Defense metabolic pathways. (A) Phenylpropanoids and lignin pathways (B) Diterpenoids pathway and (C) Benzoxazinoids pathways.

Red rectangles are upregulated, green rectangles represent downregulated genes, and black ones are non-responsive to hrip1 treatment.


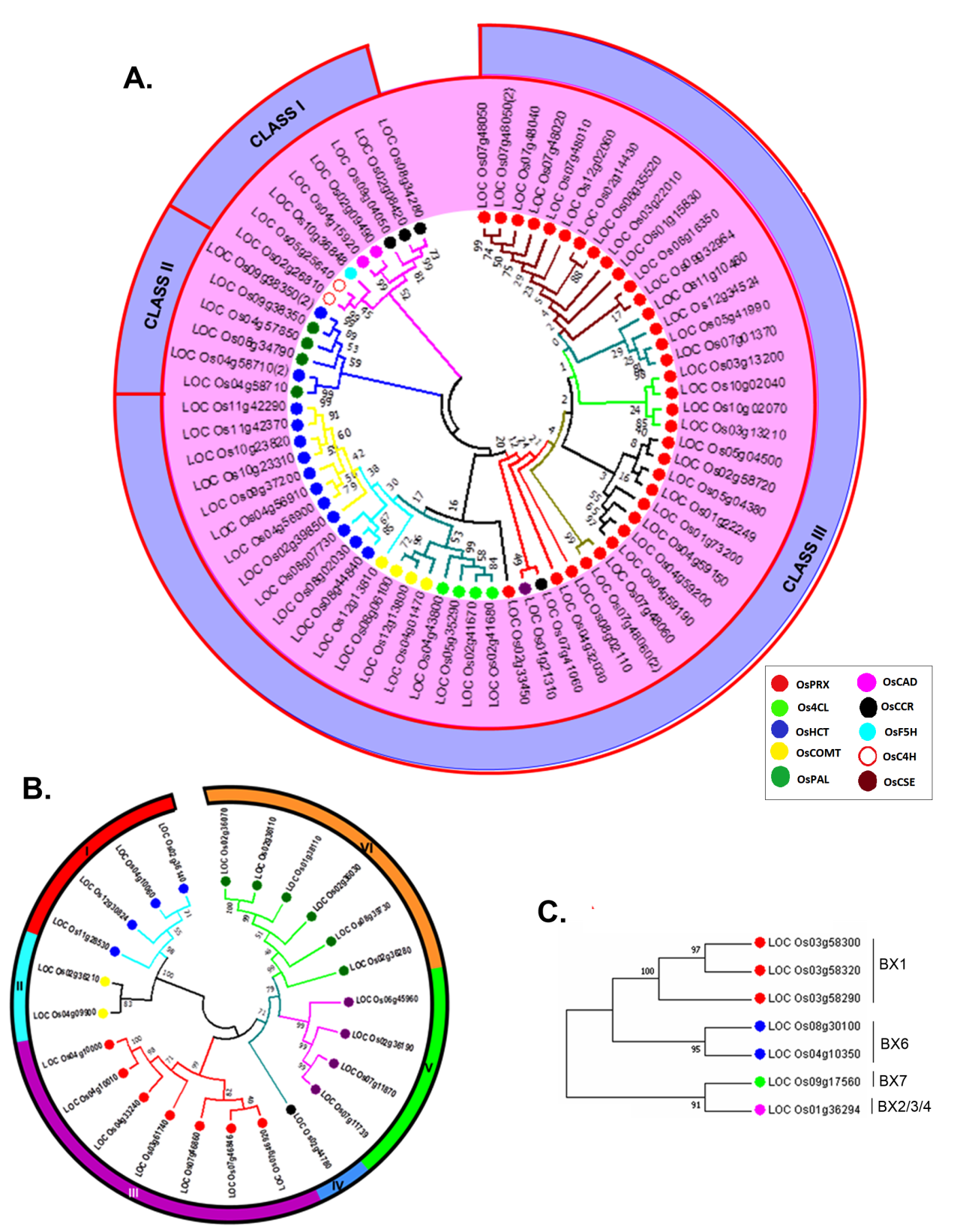


**Supplemental Figure 5: Phylogeny analysis of induced pathway genes**

(A). Phylogenetic of tree lignin biosynthetic genes (B) Phylogenetic analysis diterpenoids biosynthetic genes. I = Kaurene synthase or class I diterpene synthases (KSL), II = ent- and syn-copalyl diphosphate or class II diterpene synthases (CPS), III = Momilactone synthases, IV = Geraylgeranylediphosphate precursor (GGPP), V = Cytochrome P450 subfamily (CYP71Z), VI = Cytochrome P450 subfamily (CYP70M) (C) A phylogenetic tree illustrating the functional relatedness of BX genes

1. **†**Corresponding author. Hongmei Zeng, E-mail: [zenghongmei@caas.cn](mailto:zenghongmei@caas.cn)

   Funding agency: The National Key Research and Development Program of China, Grand number: 2017YFD0200900 [↑](#footnote-ref-1)
